# Decreased prevalence of sepsis but not mild or severe *P. falciparum* malaria is associated with pre-existing filarial infection

**DOI:** 10.1186/1756-3305-6-203

**Published:** 2013-07-10

**Authors:** Madhumita Panda, Prakash K Sahoo, Alok Das Mohapatra, Soumya kanti Dutta, Pravat K Thatoi, Rina Tripathy, Bidyut K Das, Ashok K Satpathy, Balachandran Ravindran

**Affiliations:** 1 Infectious Disease Biology group, Institute of Life Sciences, Bhubaneswar, Odisha, India; 2 Division of Immunology, Regional Medical Research Centre, Bhubaneswar, Odisha, India; 3 Department of Internal Medicine, SCB Medical College, Cuttack, Odisha, India; 4 Department of Biochemistry, SCB Medical College, Cuttack, Odisha, India

**Keywords:** Coinfection, Filariasis, Severe malaria, Sepsis, *P. falciparum*, Regulatory T cells

## Abstract

**Background:**

Enhanced inflammatory host responses have been attributed as the cellular basis for development of severe malaria as well as sepsis. In contrast to this, filarial infections have been consistently reported to be associated with an immunological hypo-responsive phenotype. This suggests that successful control of filariasis by employing mass drug administration, could potentially contribute to an increase in incidence of sepsis and cerebral malaria in human communities. A case control study was undertaken to address this critical and urgent issue.

**Methods:**

Eighty-nine patients with sepsis and one hundred and ninety-six patients with *P. falciparum* malaria all originating from Odisha, were tested for prevalence of circulating filarial antigens - a quantitative marker of active filarial infection. Antibodies to four stage specific malarial recombinant proteins were measured by solid phase immunoassays and circulating CD4+CD25^high^ T-cells were quantified by flow cytometry with an objective to study if pre-existing filarial infections influence antibody responses to malarial antigens or the levels of circulating T-regulatory cells in *P. falciparum* infected patients.

**Results:**

Prevalence of filarial antigenemia was significantly less in sepsis patients as compared to controls suggesting that pre-existing filariasis could influence development of sepsis. On the other hand, levels of circulating filarial antigen were comparable in severe malaria cases and healthy controls suggesting that development of severe malaria is independent of pre-existing *W. bancrofti* infections. Plasma TNF-a, RANTES and antibodies to recombinant malarial proteins as well as levels of circulating CD4+ CD25^high^ cells were comparable in malaria patients with or without filarial infections.

**Conclusions:**

These observations imply that successful control of filariasis could have adverse consequences on public health by increasing the incidence of sepsis, while the incidence of severe malaria may not adversely increase as a consequence of elimination of filariasis.

## Background

Lymphatic dwelling filarial parasites cause severe morbidity in human hosts and persist for long durations in infected hosts. The Global Programme to Eliminate Lymphatic Filariasis (GPELF) is currently targeting elimination of the disease through annual mass drug administration (MDA) of albendazole with either DEC or ivermectin. This has been widely acclaimed to be one of the successful public health programmes and is expected to block transmission of filariasis in endemic countries by 2020 [1].

Sepsis is one of the major causes of mortality around the world and malaria is considered to be one of the most severe infectious diseases afflicting the world’s most impoverished populations. Severe malaria presents itself with a range of biological dysfunctions, i.e. anemia, respiratory complications, acidosis, renal failure, pulmonary edema, multi-organ failure and cerebral malaria etc. [2]. Development of sepsis and severe malaria share a common biology with activation of uncontrolled inflammatory host responses being the cellular and molecular basis for clinical manifestation. Both diseases are associated with elevated plasma levels of TNF-a, IL-6, IL-1β, etc. [3,4]. TNF-a is produced and released by host cells following exposure to various malarial antigens. The increase of TNF-a release is responsible for the overexpression of adhesion molecules, hence influencing sequestration of parasitized RBCs [5]. Similarly RANTES (Regulated on Activation Normal T-Cell Expressed and Secreted) is a chemokine involved in the generation of inflammatory infiltrates. Recent studies indicate that degradation of cell-cell junctions, blood–brain barrier dysfunction, recruitment of leukocytes and *Plasmodium*-infected erythrocytes and occlusion of microvessels are associated with RANTES expression. Additionally, activated lymphocytes, platelets and endothelial cells release large quantities of RANTES, suggesting a unique role for RANTES in generation and maintenance of the malaria-induced inflammatory response [6]. Low levels of RANTES correlate with disease severity and mortality in individuals with sepsis [7]. However, data on the role of RANTES in malaria appears to be contradictory. Decreased plasma levels of RANTES were documented in children with severe malarial anemia [8], but in another study, increased mRNA expression of RANTES was found in the brains of children who died of CM [9]. One of the probable reasons for low levels of RANTES in severe malaria may be the thrombocytopenia commonly associated with this condition, as platelets are major reservoirs of RANTES in peripheral circulation [10]. Systemic nematodes on the other hand are known to down regulate such responses in infected hosts [11]. This later phenomenon has been attributed to production of an array of immunomodulatory molecules released by helminthes that skew host responses away from a pro-inflammatory phenotype [12]. These observations suggest that pre-existing helminth infections could influence development of sepsis or malaria in a given host. Prevalence of cerebral malaria has been reported to be low in children with *Ascaris lumbricoides* infection, suggesting that endemic subjects harboring helminthic infections could become protected against development of cerebral malaria [13]. Animal models of sepsis and cerebral malaria have been used to address the issue, although such models do not truly represent the human disease. Concomitant infection with *S. mansoni* and *P. berghei* ANKA infection has been reported to lead to reduced cerebral manifestations [14]. More recently, it has been demonstrated that filarial parasite induced secretion of IL-10 is responsible for developing resistance to murine cerebral malaria [15], although this does not appear to be a consistent feature since observations to the contrary have also been reported [16]. For example, in a study on co-infection of mice with *Litomosoides sigmodontis* and *P. chabaudi*, mice free of circulating microfilaria were shown to develop more severe forms of malaria than animals free of microfilaria [16].

Here we report a case control study in two cohorts of patients, one with clinically proven sepsis and the other with severe *P. falciparum* malaria and quantified circulating filarial antigen (CFA), to test the hypothesis whether pre-existing filarial infections could influence development of severe malaria or sepsis. Insights into this aspect are of critical public health importance in predicting possible outcomes of the ongoing successful filariasis control programme on the incidence of sepsis or severe malaria in human populations.

## Methods

### Study area & subject recruitment

Patients with symptoms of sepsis admitted to the Department of Medicine at S.C.B. Medical College were classified into three categories. 1) Sepsis (n=36): Infection, documented or suspected, with signs and symptoms of an inflammatory response**,** viz., leukocytosis or leucopenia, increased C-reactive protein, increased procalcitonin levels. 2) Severe sepsis (n=24): Sepsis complicated by multi-organ dysfunction. 3) Septic shock (n=29): Severe sepsis with acute circulatory failure characterized by persistent arterial hypotension despite adequate volume administration. Details of sepsis patients are shown in Table 1. For classifying patients and determining outcomes, the Acute Physiology and Chronic Health Evaluation II (APACHE II) scoring system was used [4].

**Table 1:** Prevalence of sepsis in filariasis infected subjects

**Subjects**	**Sepsis (n=89)**		**Healthy control (n=38)**		**P value**
	**CFA+ve**	**CFA-ve**	**CFA+ve**	**CFA-ve**	
**Number (%)**	*6 (6.7)	83 (93.3)	*16 (42.2)	22 (57.8)	CFA+ve in sepsis vs HC (<0.0001), CFA-ve vs in sepsis vs HC(<0.0001)
**Sex(M/F)**	6/0	55/28	9/7	10/12	-
**Mean Age in years(range)**	42.83(24–73)	46.24(17–85)	31.22(20–75)	29.85(12–69)	-
**APACHE II score**	14.25 ± 4.15	12.63 ± 0.58	-	-	CFA+ve vs CFA-ve, NS
**Mechanical ventillaion**	2 (33.34)	7 (8.43)	-	-	CFA+ve vs CFA-ve, NS
**Vasopressors**	2 (33.34)	18 (21.6)	-	-	CFA+ve vs CFA-ve, NS
**Total leukocyte count (10**^**3**^**/mm**^**3**^**)**	13.4-30	7.2-150	ND	ND	CFA+ve vs CFA-ve, NS
**CRP (mean±SD) mg/ml (Range)**	86.92 ± 34.10 (16.33-146.6)	97.17±5.18 (10.42-150.8)	ND	ND	CFA+ve vs CFA-ve, NS

NOTE: CFA; circulating filarial antigen.*p value<0.0001, 95% Confidence Interval, Odds ratio = 0.099. Data are no. (%) of participants unless otherwise specified.

Patients reporting at the out-patient department and/or admitted to the Department of Internal Medicine at S.C.B. Medical College (Cuttack, India), with a short history of fever associated with unarousable coma or multi organ dysfunction were clinically assessed. Patients with microscopically demonstrable *P. falciparum* in thick blood smears were recruited for the study. Diagnosis by microcopy was further confirmed by immuno-chromatographic card test. Details of malaria patients are shown in Table 2. Non-complicated malaria (NCM) was defined as patients reporting to the outpatient department with fever and evidence of *P. falciparum* infection. Patients classified with severe malaria belonged to one of the following three groups:1) Cerebral malaria (CM, n=48), 2) Non cerebral severe malaria (NCSM, n=13) and 3) Multi-organ-dysfuction (MOD, n=64) [17]. Thirty-eight normal subjects of comparable ethnicity and originating from the same areas as that of patients and free of demonstrable malarial infections were taken as healthy controls. The current studies on sepsis and malaria were approved by the Ethics Committee of SCB Medical College and blood samples were collected after obtaining written consent from patients or accompanying persons.

**Table 2:** Details of study participants

**Subjects**	**SM**	**NCM**	**HC**
**Total number (n)**	125	71	38
**Sex(M/F)**	96/29	47/24	19/19
**cp**	34(15–72)	30(14–70)	30(12–75)

NOTE: *SM* Severe malaria1, *NCM* Non- complicated malaria, *HC* Healthy controls.

### Flow cytometry

About 5ml of the venous blood was collected in heparin from patients, plasma was separated and frozen at −20°C until further use. 100 ul of whole blood was used for two color staining with PE-cy5 labelled anti-CD4 and FITC labelled anti-CD25 (BD Biosciences), along with appropriate isotype controls. Stained cells were then acquired on a 2-laser/4 channel BD FACS Calibur Flow Cytometer and analysed using CellQuest Pro Software.

### Enzyme-linked immunosorbent assay (ELISA)

Plasma concentrations of TNF-a and RANTES were estimated using commercial sandwich ELISA kits (Sanquin, Amsterdam) according to the manufacturer’s instructions. Circulating Filarial Antigens (CFA) were measured by Trop Bio ELISA test kit (Trop Bio Pvt Ltd, Townsville, Australia) as described earlier by us [18]. Antibodies to malarial recombinant proteins, by solid phase assay using sporozoite surface protein (SSP-2), circum-sporozoite protein (CSP), exported antigen AG5.1 (Exp-1) and liver stage antigen-1 (LSA-1) of *P. falcipaarum*. Sera were tested after 200 fold dilutions and bound antibodies were detected using 1000-fold-diluted HRP-labeled anti-human IgG (P0216; Dako) and enzyme activity was measured using OPD and absorbance read at 492 nm. The results were expressed as arbitrary ELISA units, using internal laboratory standards.

### Statistics

**Figure 1:**
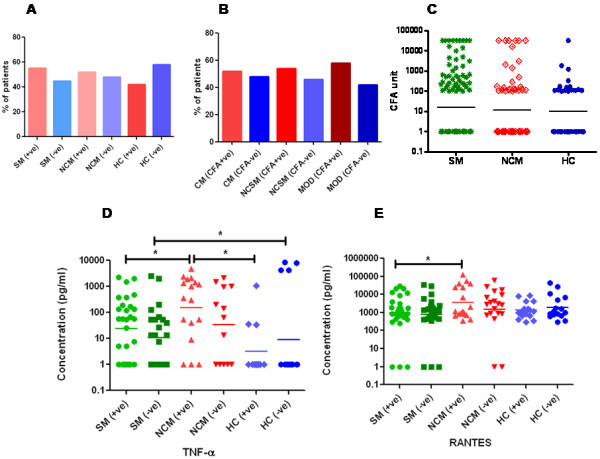
**Prevalence of circulating filarial antigen, Levels of CFA and levels of plasma cytokines in malaria patients.** Number of severe malaria, non-complicated malaria and healthy control individuals with and without active filarial infection is shown. The sample numbers are SM (CFA+ve= 69, CFA-ve=56), NCM (CFA+ve=37, CFA-ve=34) and HC (CFA+ve=16, CFA-ve=22). Fisher’s-exact test was employed to compare percentages between groups **(A)**. Number of cerebral malaria, non-complicated severe malaria and individuals with multiorgan dysfunction with and without active filarial infection is shown. The sample numbers are CM (CFA+ve= 25, CFA-ve=23), NCSM (CFA+ve=7, CFA-ve=6) and MOD (CFA+ve=37, CFA-ve=27). Fisher’s-exact test was employed to compare percentages between groups **(B)**. Circulating filarial antigen levels are comparable in SM (n=125, stars), NCM (n=71, squares) and HC (n=38, circles) cases **(C)**. Association within groups was analysed by one way analysis of variance (ANOVA) followed by Tukey’s post test. Serum levels of TNF- a **(D)** and RANTES **(E)** in CFA +ve and CFA-ve were comparable in severe malaria, non-complicated malaria and healthy control individuals. Sample numbers for TNF-a: SM (CFA+ve=30, CFA-ve=25), NCM (CFA+ve=17, CFA-ve=13) and healthy controls (CFA+ve=12, CFA-ve=16). Sample numbers for RANTES: SM (CFA+ve=32, CFA-ve=26), NCM (CFA+ve=17, CFA-ve=18) and healthy controls (CFA+ve=15, CFA-ve=15). P values were determined by unpaired student’s t-test.

**Figure 2:**
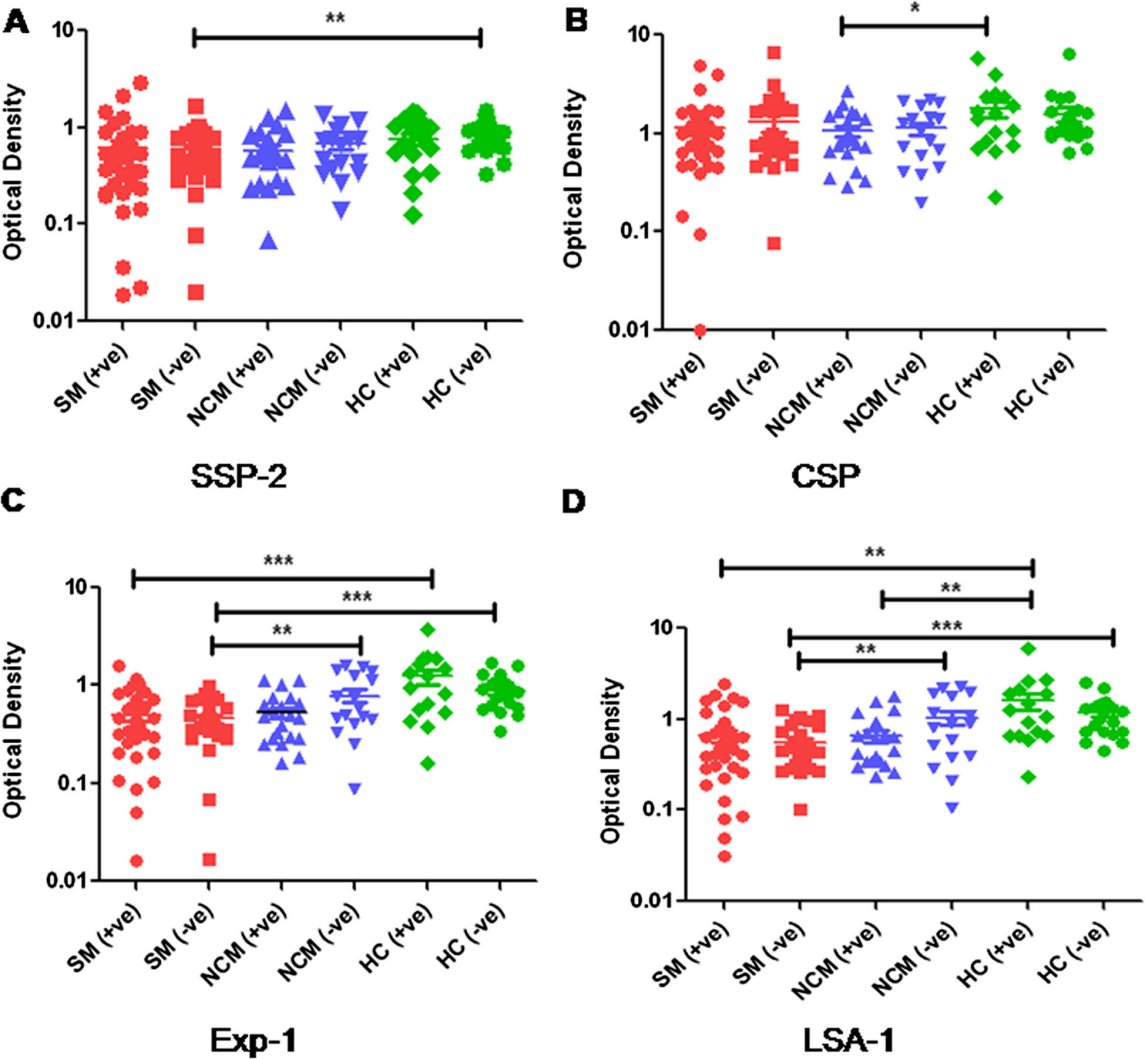
**IgG antibody titers to recombinant malarial antigens in SM, NCM and healthy control individuals.** IgG Antibodies to recombinant malarial proteins SSP-2 **(A)**, CSP **(B)**, Exp-1**(C)** & LSA-1 **(D)** among CFA+ve, CFA-ve patients in SM (red, CFA+ve=35, CFA-ve=26), NCM (blue, CFA+ve=21, CFA-ve=17) and HC (green, CFA+ve=16, CFA-ve=22) as determined by ELISA are shown. Each symbol represents antibody levels in individual samples and horizontal bars represent the Geometric mean for each group. Students’ unpaired t-test was used to analyze differences in levels among groups. No significant differences were observed between the three groups.

**Figure 3:**
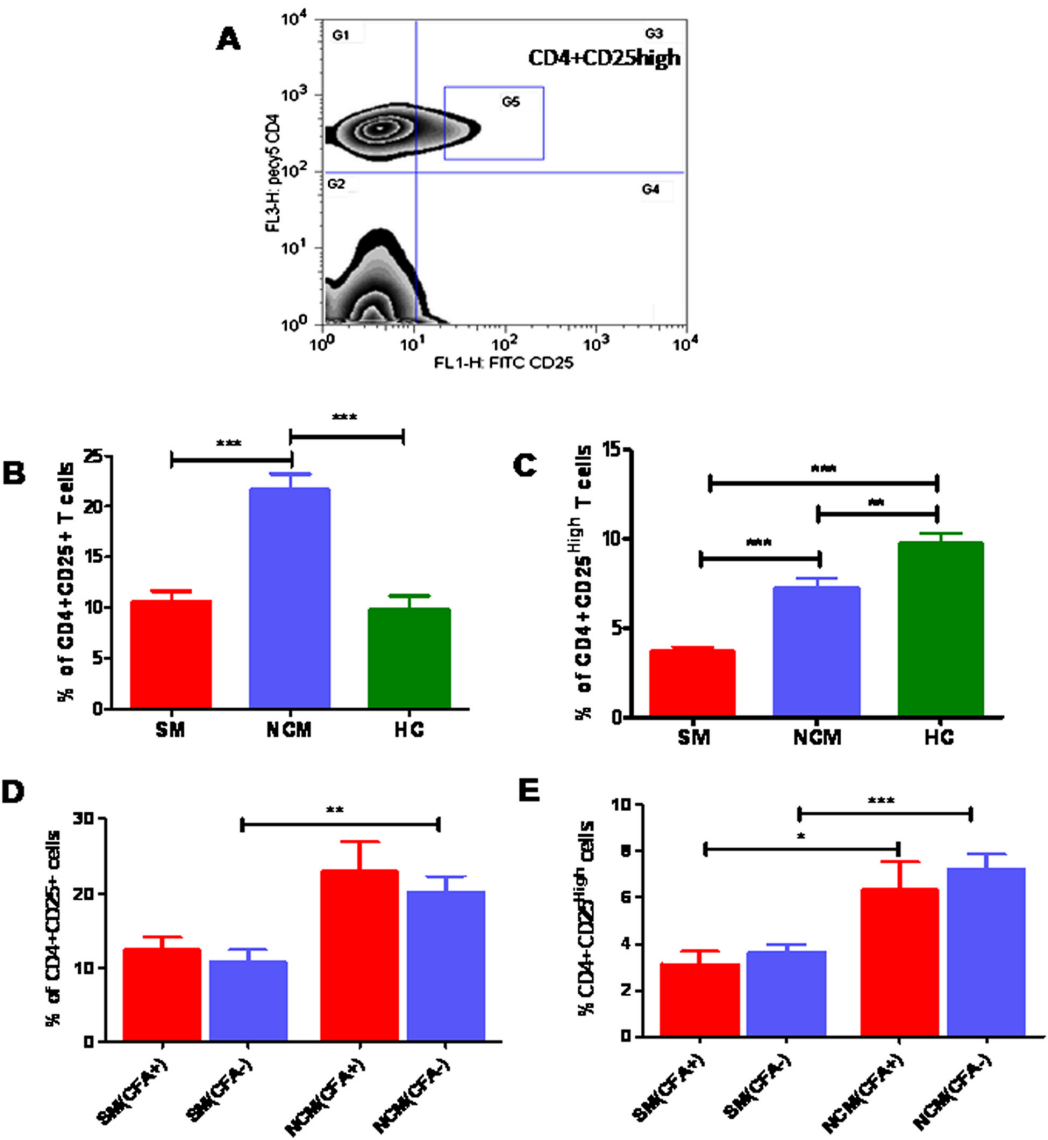
**Profile of CD4+CD25+ve cells in malaria patients with and without CFA.** PBMCs of study subjects at the time of recruitment were analyzed by flow-cytometry for CD4 and CD25 expression after staining with anti-human CD4-PE.cy5 and anti-human CD25-FITC. Percentage of CD4+ve T cells expressing CD25 are shown here, **A**: Zebra plot for CD4+CD25^high^ T–cells, G1 represents CD4+cells, G4 represents CD25+cells, and G3 represents CD4 + CD25+ cells and G5 represents CD4+CD25 high cells. Percentage of CD4+CD25+ T cells **(B)** and CD4+CD25^high^ T **(C)** cells in SM=91, NCM=58 and control=22 are shown. The association within the groups was analysed by one way analysis of variance (ANOVA) followed by Tukey’s post-hoc test. Percentages of CD4+CD25+ve T cells and CD4+CD25^high^ T cells in severe malaria (CFA+ve=14, CFA-ve=64), non-complicated malaria (CFA+ve=13, CFA-ve=34) patients with or without active filarial infection are shown in **D** and **E** respectively. The association within the groups were analysed by one way analysis of variance (ANOVA) followed by Tukey’s post- hoc test.

Statistical analyses were performed by using GraphPad Prism (version 5.01). For analysis of Figure 1(D and E) and Figure 2 (A, B, C and D) unpaired student’s t test was applied to determine differences between groups. For analysis of Figure 1A, B and Table 1, Fisher-exact test was employed to calculate Odds ratios (ORs) at 95% confidence intervals. For analysis of Figure 1C and Figure 3 (B, C, D and E) the association within the groups were analysed by one way analysis of variance (ANOVA) followed by Tukey’s post-hoc test. *P*-values<0.05 were considered to be statistically significant.

## Results

### Prevalence of filariasis in sepsis and malaria patients

Prevalence of circulating filarial antigen (CFA) was significantly less in patients with sepsis in comparison to healthy endemic controls (Table 1). While 42.2% of controls were found to harbor CFA, only 6.7% of patients with sepsis were positive for antigenemia with relatively low levels of antigenemia (CFA levels of the six CFA+ve patients were 154, 398, 625, 154, 354 & 542 respectively). These findings suggest that pre-existing filarial infections could be preventing development of clinical sepsis (Table 1). On the other hand prevalence of filarial antigenemia was comparable in severe malaria patients, non complicated malaria and healthy controls (Figure 1A). Similarly, the prevalence of filarial antigenemia was also comparable in three subgroups of severe malaria i.e. cerebral malaria, non-cerebral severe malaria and multiorgan dysfunction (Figure 1B). About 45-50% of patients in all the groups were found to harbor filarial antigenemia and no statistically significant association was observed between severe malaria (SM), non-complicated malaria (NCM) and healthy controls, suggesting that pre-existing filarial infections neither predispose nor offer resistance for development of severe malaria. The CFA levels were comparable in the three categories further validating the above conclusion (Figure 1C). Similarly, levels of TNF-a as well as RANTES were also comparable in SM and NCM patients with and without filarial antigenemia (Figure 1D and E), suggesting that induction of these inflammatory molecules in *P. falciparum* malaria is not influenced by pre-existing filarial infection. However, TNF-a was significantly higher in SM cases in comparison to healthy controls (data not shown). Significantly higher levels of RANTES were observed in NCM patients in comparison to SM patients suggesting their importance during non-complicated malarial infection (Figure 1E).

### Plasma Ab titers against malaria Ags

Specific IgG antibody titers to four different *P. falciparum* recombinant proteins were quantified in SM, NCM patients and healthy controls and the data for SSP-2, CSP, Exp-1 and LSA-1 are shown in Figure 2A, B, C and D. No significant difference in IgG antibody titers were observed among CFA+ve and CFA-ve cases of SM, NCM and healthy controls suggesting that humoral immune responses to these malarial proteins was not significantly altered as a consequence of pre-existing filarial infections. IgG to Exp-1 and LSA-1 was significantly low in SM patients in comparison to healthy controls in CFA+ve and CFA-ve cases (Figure 2C & D). Similarly IgG to CSP was significantly low in CFA+ve cases of NCM patients in comparison to CFA+ve cases of healthy control group (Figure 2B). On the other hand serum levels of IgG to SSP-2 were significantly low in SM patients in comparison to healthy controls (Data not shown), but when both the groups were further sub-divided as CFA+ve and CFA-ve, a significant difference was observed only among CFA-ve cases of both groups. Antibody levels to malarial recombinant proteins were significantly more in healthy controls in comparison to patients with acute malaria. Similar observations of decreased levels of malarial antibodies in patients have been reported by others [19], which has been attributed to the presence of circulating malarial antigens and formation of immune-complexes. IgG subtypes i.e. IgG1, IgG2, IgG3 or IgG4 specific to malaria recombinants may have revealed differences between CFA+ve and –ve subjects. However, such a possibility could not be tested due to shortage of malarial recombinant proteins.

### CD4+CD25^high^ T cells in the study population with or without active filarial infection

Filariasis and chronic malaria have been reported to influence the normal balance of immune-regulatory T lymphocytes**.** For this preliminary study CD4+CD25^high^ cells were regarded as T-regulatory cells and the gating strategy to score them is shown in Figure 3A [20]. While scoring CD4+CD25^high^ populations, we also scored CD4+CD25+ T cells and significantly higher levels of both the cell populations were observed in NCM cases compared to SM patients (Figure 3B and C). However, when SM, NCM and healthy controls are analyzed in the context of filarial antigenemia, no significant difference in levels of this T cell phenotype was observed between the groups indicating that levels of circulating CD4+CD25+ and CD4+CD25^high^T cells in malaria patients are not influenced as a consequence of pre-existing filarial infection (Figure 3D and E).

## Discussion

The primary objective of this case–control study was to address a critical public health issue viz., what will be the consequences of an ongoing successful Filariasis control programme on the incidence of severe malaria or sepsis in human communities? The study was conducted in a tertiary hospital in eastern India that reports high incidence of all three diseases. The observations on incidence of severe malaria vis-à-vis filarial antigenemia should come as a relief to public health professionals since the data suggests that incidence of severe manifestations of *P.falciparum* malaria such as cerebral malaria, multi-organ dysfunction etc., may not adversely increase as a consequence of control/elimination of filariasis in human communities. These results do not appear to be in consonance with several co-infection studies conducted in experimental models of severe malaria and nematode infections such as *L. sigmodontis*[16]*, H. polygyrus*[21]*or B. pahangi*[15]. Co-infection experiments conducted in animals, however, need to be interpreted with caution since a) animal models of cerebral malaria are not considered as truly representative of severe malaria in humans [22], b) use of genetically defined strains of mice do not reflect genetic heterogeneity observed in human populations and more importantly c) such studies do not factor-in epigenetic parameters and other common variables observed in human communities.

Our finding on significantly low prevalence of sepsis in subjects with filarial antigenemia suggests that development of clinical sepsis (a hyperinflammation state) is prevented by pre-existing chronic filarial infections. This is analogous to another report in the literature in which prevalence of chronic inflammatory diseases such as type1 diabetes was less frequent in subjects who were positive for filarial antigenemia [23]. Recently we also reported absence of filarial antigenemia in rheumatoid arthritis patients living in filarial endemic areas [24]. These observations give credence to our earlier proposal that circulating filarial antigens bind to TLR-4 and block activation of human monocytes by endotoxin. We have also previously demonstrated that a filarial glycoprotein activates murine macrophages and human monocytes *in vitro* in a non-inflammatory pathway and also blocks development of endotoxemia in mice. LPS failed to activate PBMCs of subjects with active filarial infection as shown by significantly low levels of synthesis and release of inflammatory cytokines *in vitro*[25]. A somewhat similar scenario has been reported in human Schistosomiasis also further suggesting that systemic nematode infections could be offering resistance to hosts from developing clinical sepsis [26]. The above studies are further supported by a paper published by Hubner *et al*. in Litomosoides sigmodontis, which suggests that adult worms suppress LPS induced endotoxemia [27]. However, since the population in the studied region is covered by the MDA programme of DEC and albendazole, it is possible that the infected patients are positive only for antigenemia (CFA) and not for microfilaraemia and thus are protected from sepsis. It has been widely proposed that as a consequence of significant decrease or elimination of infectious diseases in economically developed countries there has been an increase in incidence of allergy and autoimmune diseases over the last century [28]. Low prevalence of filarial antigenemia in sepsis patients observed in this study tends to support the notion that increased incidence of sepsis in developed nations during the last 100 years could be due to elimination of metazoan pathogens in these countries. If this is true the results of the current study suggest that success of the Global drive for filariasis elimination could have adverse consequences by way of increasing incidence of sepsis. Validation of this conclusion is an urgent requirement and emphasizes the need to undertake similar investigations on filarial infections in sepsis patients in other geographical locations and to arrive at a clearer and more conclusive portrait of the consequences of elimination of filariasis. The current study, however, has two minor limitations; a) prevalence of geo-helminthic infection, which might influence severity of malaria, was not scored due to non-availability of stool samples from comatose patients or from sepsis patients in ICUs. However, the prevalence of soil transmitted helminthes in India is available in (Report of the Informal Consultation on Scaling up Treatment of Soil Transmitted Helminthiasis in the South-East Asia Region, 2011(26) a WHO report on a survey of soil transmitted helminthes in India, which shows the prevalence rates from 0.5% to 42% between 1999 to 2003. b) circulating microfilaria could not be quantified. Both these limitations were due to ethical as well as practical considerations since patients in ICUs cannot be subjected to nocturnal blood or stool sample collection. However, the second issue is not very critical since circulating microfilaria are rapidly eliminated by single annual MDA which does not influence antigenemia, taken as a basis of filariasis in the current study.

## Conclusions

The above study suggests that incidence of severe malaria may not adversely increase as a consequence of filariasis elimination programme in human communities. Furthermore a significantly low prevalence of sepsis in subjects with filarial antigenemia was observed which suggests that development of clinical sepsis (a hyperinflammation state) is prevented by pre-existing chronic filarial infections.

## Competing interests

The authors declare that they have no competing interests.

## Authors’ contributions

MP, ADM and PS carried out the experiments. MP, ADM wrote and drafted the manuscript. BKD supplied the blood samples and performed clinical categorization of patients. AKS contributed to analysis of data. BR conceived the idea, designed the experiment and finalized the manuscript. All authors read and approved the manuscript.
